# Weight Is More Accurate than Gestational Age When Estimating the Optimal Endotracheal Tube Depth in Neonates

**DOI:** 10.3390/children8050324

**Published:** 2021-04-22

**Authors:** Hsien-Kuan Liu, Yung-Ning Yang, Shu-Leei Tey, Pei-Ling Wu, San-Nan Yang, Chien-Yi Wu

**Affiliations:** 1 Department of Pediatrics, E-DA Hospital, Kaohsiung 82445, Taiwan; hkleda0608@gmail.com (H.-K.L.); ancaly@yahoo.com.tw (Y.-N.Y.); djsr2000@hotmail.com (S.-L.T.); peiling0420@gmail.com (P.-L.W.); y520729@gmail.com (S.-N.Y.); 2 School of Medicine, I-Shou University, Kaohsiung 82445, Taiwan

**Keywords:** body weight, endotracheal tube, gestational age, intubation, neonate, resuscitation

## Abstract

Determining the optimal endotracheal tube (ETT) depth in neonates remains challenging for neonatologists. The guideline for optimal ETT depth is based on the patients’ weight or gestational age. However, there is a discrepancy in the suggested ETT depth between these two parameters. The aim of this retrospective study was to compare the recommended weight-based and age-based formulas for optimal ETT depth and obtain the optimal reference before intubation. Participants were assigned to group 1 if the recommended ETT insertion depth based on weight was concordant with the recommended depth based on gestational age, and to group 2 if the weight and age-based depth recommendations were discordant. After exclusion, 180 patients were included in the analysis. Results indicated that the predicted ETT depth suggested by age required more adjustment than by weight (*p* < 0.05). Furthermore, the required adjustment in the weight-based formula was smaller than the age-based formula (*p* < 0.05). Multivariate linear regression analysis revealed that weight was the key factor affecting the optimal depth (*p* < 0.001). These results imply that when there is a discrepancy in ETT depth between the weight-based and age-based recommendation, the weight-based one will be more accurate than the age-based one.

## 1. Introduction

Predicting the optimal depth of the endotracheal tube (ETT) in intubated neonates remains challenging for neonatologists. This measurement is crucial for neonates to achieve adequate respiratory support and for surfactant delivery. In addition, a malpositioned ETT can result in adverse outcomes in this vulnerable population. An ETT that is too deep could result in complications such as pneumothorax, desaturation, and lung collapse, while an ETT that is too shallow could lead to accidental extubation [1,2,3]. However, the difference between the optimal ETT depth and the depth on the first attempt is usually within a range of less than 1 cm. Consequently, how to correctly place the ETT on the first attempt and minimize later adjustments is a considerable issue for pediatricians.

The recommended suggestions for ETT depth vary from Tochen’s formula ((Body weight (kg) + 6) centimeters) [4,5] to the latest age- and weight-based ETT depth calculation formulas from the Textbook of Neonatal Resuscitation, 7th edition [6], which has been fully implemented since 2017 in Taiwan. We adhere to the textbook guide using both age and body weight to determine the depth of the ETT on the initial insertion attempt. The recommended ETT depth by the 7th Neonatal Resuscitation Program (NRP) is the tip of the tube located in the mid-trachea adjacent to the first or second thoracic vertebra. A depth outside this suggestion is considered to be malposition. However, it is common to see a discrepancy in the suggested ETT depths based on body weight or age. A discrepancy in ETT depth can be seen with different age-based or weight-based recommendations in the 7th NRP. For example, the suggested insertion depth for a 1500 g preterm baby with a gestational age of 35 weeks would be 8.0 cm based on age and 7.5 cm based on body weight according to the 7th NRP, respectively (Table 1) [7]. When a discrepancy exists, choosing the appropriate insertion depth poses a dilemma.

There are few reports in the literature discussing this issue. When neonates need to be intubated, their poor respiratory status requires immediate support. Even minor adjustment of the ETT cannot be tolerated in this critical situation. To resolve the dilemma for intubation of patients with inconsistent age and weight recommendations, we designed a study to compare the accuracy of ETT depths based on each parameter.

## 2. Materials and Methods

### 2.1. Study Design and Subjects

In this retrospective study, we reviewed the records of neonates admitted to the neonatal intensive care unit (NICU) of E-Da Hospital, Kaohsiung, Taiwan, from June 2013 to June 2019. Ethics approval was obtained from the E-Da Hospital Institutional Review Board (EMRP-109-032). Patient information was de-identified before analysis, and informed consent was waived. Records for neonates who underwent intubation were reviewed. Neonates who were intubated immediately after birth or intubated at a postmenstrual age of less than 44 weeks were included in this study. Chest X-ray was performed after intubation in all patients. Neonates with congenital anomalies and incomplete medical records were excluded. Moreover, neonates with gestational age less than 23 weeks or body weight less than 500 g were also excluded due to a lack of recommendations in the 7th NRP. General characteristics data (gestational age, postmenstrual age on intubation, body weight, gender, body length, Apgar score, and mode of delivery), final ETT depth, and depth of adjustment were documented. The intubations were performed by neonatologists or experienced neonatology fellows. The neonates were stratified into 2 groups. Group 1 included neonates for whom the recommended 7th NRP weight-based and age-based ETT insertion depth were identical. Group 2 included neonates for whom there was a discrepancy in the estimated ETT depth between age-based and weight-based recommendations. For example, the recommended depth of the ETT is 8.0 cm regardless of using the weight-based or age-based formula as a reference in a 35-week-old newborn weighing 2200 g. Participants with such consistent ETT depth estimates were included in group 1. On the contrary, for a 35-week-old newborn with a body weight of 1500 g, the recommended 7th NRP weight-based and age-based ETT depths were 8.0 cm and 7.5 cm, respectively. These neonates were registered in group 2.

### 2.2. Depth of Endotracheal Tube

**Figure 1:**
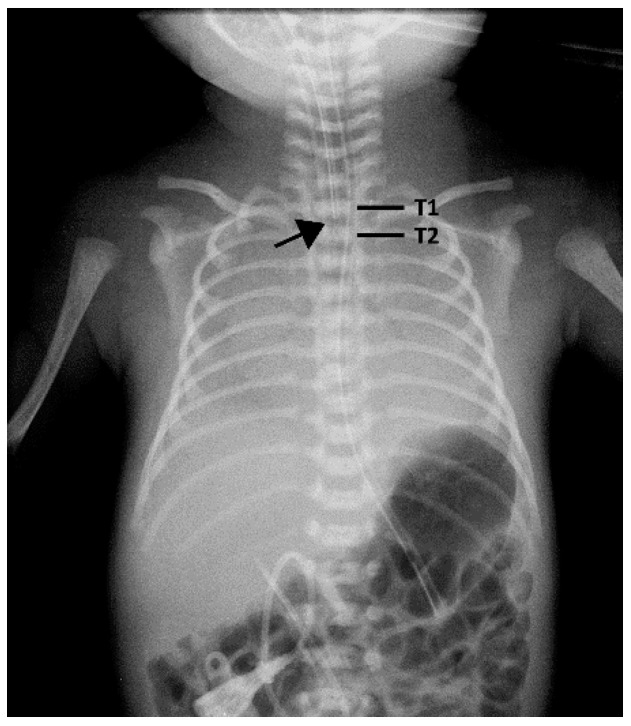
Black arrow pointing to the optimal ETT depth. (The tip of the tube is located in the mid-trachea adjacent to the first or second thoracic vertebra as recommended by the 7th Neonatal Resuscitation Program).

Before 2016, guidelines for ETT insertion depth were based on the baby’s body weight, using the following formula: ((Body weight (kg) + 6) cm) [4,5]. For example, a 2 kg infant would have an ETT depth of 8 cm ((2 + 6) cm). Starting in 2017, ETT depth was calculated in accordance with the recommendation from the Textbook of Neonatal Resuscitation, 7th ed [6]. Auscultation was performed in all neonates after intubation to confirm the ETTs were in the trachea. The final ETT depth was adjusted and confirmed after portable antero-posterior chest X-ray was performed, with the head set in the neutral position. The optimal ETT depth was defined as the tip of the tube located in the mid-trachea adjacent to the first or second thoracic vertebra according to the 7th NRP (Figure 1). All radiographs were evaluated by neonatologists who then adjusted the ETT depth. The body weight used for calculations was prenatally estimated by ultrasound birth weight for neonates intubated urgently after birth or the most recent actual body weight obtained in the NICU before intubation.

### 2.3. Statistical Analysis

Data were analyzed using SPSS version 20 (IBM Corp., Armonk, NY, USA). Baseline characteristics were analyzed. Proportions are presented for categorical variables and median (interquartile range) are used for nonparametric continuous variables. The Mann–Whitney U test (for continuous variables) and Chi-square test (for categorical variables) were used for comparing variables between the two groups. Multivariate linear regression was conducted to adjust for potential variables associated with the final optimal ETT depth.

## 3. Results

**Figure 2:**
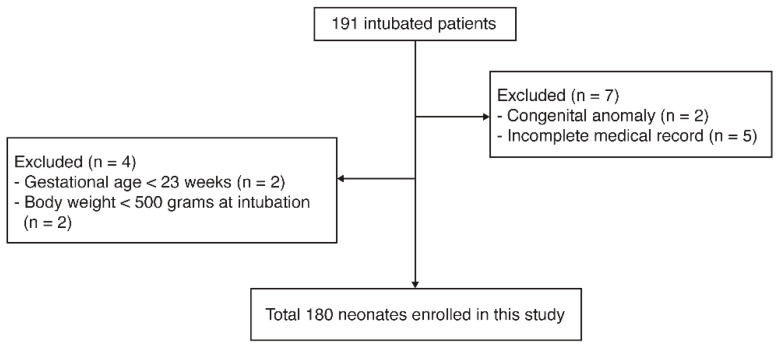
Study flowchart.

A total of 191 neonates underwent intubation during the study period. Neonates with congenital anomalies (*n* = 2) and incomplete medical records (*n* = 5) were excluded. Neonates under 23 weeks gestational age (*n* = 2) or under 500 g body weight (*n* = 2) were also excluded due to the lack of guidelines for ETT depth in the Textbook of Neonatal Resuscitation, 7th edition [6]. Finally, 180 patients were included in the analysis (Figure 2). Among the 180 neonates, 117 (65.0%) were preterm infants. The mean gestational age was 33.66 ± 5.46 weeks, and the mean body weight was 1986.50 ± 914.45 g. Most neonates were considered to have an appropriate weight for gestational age (135, 75%), with 36 (20%) small and 9 (5%) large-for-gestational-age infants. The ETT insertion on the first attempt was correct in 66.1% of cases through the study period. Furthermore, the accuracy rate before 2016 (using Tochen’s formula) and after 2017 (calculated using the 7th NRP) was 64.6% and 67.9%, respectively. There was no significant difference between the two time periods (*p* = 0.64) (Table 2).

### 3.1. Comparison of the Consistent and Inconsistent Groups

**Figure 3:**
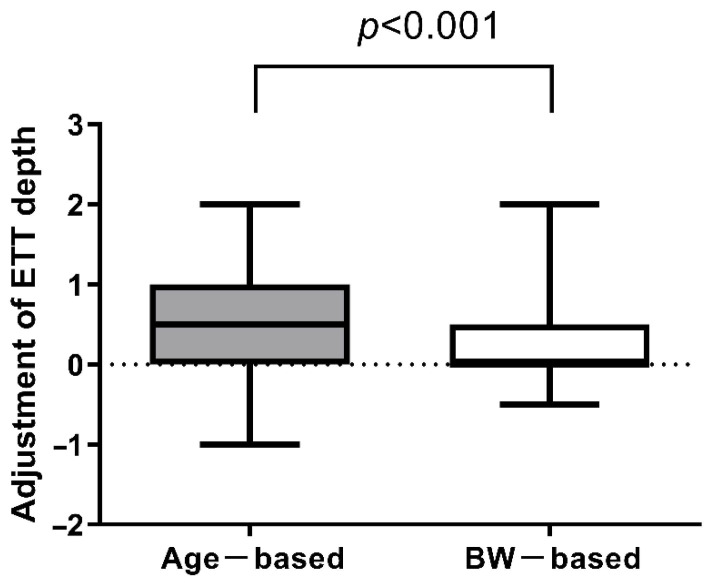
The adjusted depth for age-based and weight-based recommendations in group 2. Data are median (interquartile range). ETT: endotracheal tube; BW: body weight.

The demographic characteristics of the two groups are shown in Table 3. There was no difference in age, body weight, or body height at intubation. The depth of adjustment and percentage of appropriate for gestational age (AGA) infants did not differ significantly either. If we used age-based guidance to estimate the ETT inserted depth, the distance adjusted for was significantly greater in group 2 (0.5 (0.0–0.5) cm and 0.5 (0.0–1.0) cm, *p* < 0.05). However, there was no difference between the two groups in the adjusted depth (group 1, 0.5 (0.0–0.5) cm, and group 2, 0.0 (0.0–0.5) cm, *p* > 0.05) (Table 3) if using the body-weight-based recommendation. Within group 2, the ETT depth adjustment was significantly greater when using the age-based formula compared to the weight-based formula (0.5 (0.0–1.0) and 0.0 (0.0–0.5), respectively, *p* < 0.001) (Figure 3).

### 3.2. Multivariable Linear Regression

Potential confounding factors that might have affected the final optimal insertion depth were analyzed by multivariable linear regression and are presented in Table 4. With simple linear regression, age (gestational or post menstrual), body weight, body length, and sex were significantly associated with the final optimal depth (*p* < 0.001, <0.001, <0.001, <0.05, respectively). Nevertheless, after multivariable linear regression was performed, only body weight differed significantly (*p* < 0.001) (Table 4).

## 4. Discussion

As medical skills and nurse-to-patient ratios have advanced, the neonatal mortality rate has declined worldwide [8,9,10]. Although nasal continuous positive airway pressure is frequently applied for babies with respiratory distress, ETT insertion retains a key role for critical patients or those who fail nasal continuous positive airway pressure support [11,12,13]. An appropriate ETT depth reference is important for minimizing the frequency of adjustments, which can reduce the occurrence of complications. Numerous guidelines have been proposed for neonatologists for the accurate prediction of ETT depth to reduce the need for adjustment. Our study aimed to provide a reliable indicator for those who need to be intubated.

The optimal ETT depth has been revised over time [5,6], and several studies have re-evaluated the ideal ETT depth using new methods [5,14,15]. Since 2008, when Kempley et al. proposed that gestational age could be used to predict the optimal ETT depth more accurately than weight [7], depth based on either an age or weight formula has been recommended in the 7th NRP [6].

However, the insertion depth estimated by weight-based and age-based formulas is different, and so choosing the optimal option is an important issue. Previous studies focused on how to choose the ETT depth in small for gestational age (SGA) neonates and suggested that body-weight-based references are more accurate than gestational age-based formulas in these patients [16,17]. However, the discrepancy in the suggestions of the 7th NRP guidelines is an issue frequently encountered, not only in SGA newborns but also in AGA or large-for-gestational-age newborns. In our study, there was no difference in the proportion of AGA infants between the two groups, indicating that AGA and non-AGA neonates are equally likely to encounter the dilemma of different age- and weight-based suggested values. In addition, if we choose to use age-based recommendations for ETT tube placement, in the group where age and weight recommendations are inconsistent, the depth of adjustment required will be greater than if we choose to use weight-based recommendations (0.5 (0.0–1.0) vs. 0.0 (0.0–0.5), respectively, *p* < 0.001). However, if we used the weight-based suggestions, the adjusted distance did not differ significantly in this inconsistent group. This result implies that age-based recommendations were less accurate in the group where age and weight recommendations are inconsistent compared to the consistent recommendation group. Furthermore, weight-based suggestions could provide a more reliable prediction of optimal ETT depth. The required ETT distance revision in the inconsistent recommendation group was smaller when using the weight-based suggestions compared with the age-based. This result is in accordance with previous studies that found weight-based suggestions to be superior to age-based [16,17]. Additionally, it seems that when there is inconsistency in the weight-based and age-based ETT depth suggestion, weight-based suggestions are more accurate than age-based, for both AGA and non-AGA neonates.

Similar to our study, Flinn et al. designed a study comparing the depth correction rate for age-based and weight-based calculations [18]. In Flinn’s study, they compared the result of Tochen’s formula ((Body weight (kg) + 6) cm) to the age-based recommendation, and the results showed no significant difference between these two methods. In contrast, our study used weight-based recommendations from the new 7th NRP, which yield a more precise result than Tochen’s formula. There is scarce literature using the new weight-based ETT depth recommendation to predict the correct rate of ETT insertion, and our result might prove valuable for neonatologists.

There is a possible explanation for the association between the final optimal ETT depth and body weight. Among children and adults, the tracheal length increases with age and decreases after 70 years due to fibrous tissue reduction [19,20]. Nevertheless, few published reports discuss infant tracheal length owing to the difficulty in studying infants. Lee and Yang designed a study using video rigid ventilation bronchoscopy to estimate airway length, and they found that the tracheal length was best correlated with body weight in infants [19]. Clinically, there is a vast difference in body weight between neonates even at the same age. Since the tracheal length increases as weight increases, this indicates that weight-based ETT depth would be more precise than age-based ETT depth.

The correlation between body length and ETT depth has also been proposed in the literature [17]. Although body length, body weight, age, and sex were significantly associated with final ETT depth during simple linear regression in our study, only body weight had an influence on the final ETT depth after multiple linear regression. This result implies that body weight is the most important parameter in deciding the optimal ETT depth.

A previous study also compared the accuracy of ETT depth using the body-weight-based and age-based formula [18]. In that study, Flinn et al. compared two recommendations for ETT depth with the age-based formula and Tochen’s formula ((Body weight (kg) +6) cm). The result indicated that age compared to weight did not result in more correctly placed ETTs. In contrast, our study used the newest 7th NRP and the result indicated that the weight-based formula is more accurate than the age-based formula. When faced with the discrepant recommendation during intubation, our results provide clinicians with a good choice to decrease the chance of re-position.

The other issue worth discussing is the malposition rate in neonates who need intubation. According to previous studies, the prevalence of ETT malposition ranges from 28.9% to 57%, and in 47% of infants <1 kg [14,15,21]. The high prevalence of malposition indicated that neonates might be exposed to another dangerous situation. In patients who need intubation, this situation should be avoided as much as possible. To minimize the re-position rate, our result gives neonatologists a reliable guide to follow.

We acknowledge that this study has some limitations. First of all, it was a retrospective study performed at one institution and had a relatively small sample-size. Therefore, further large-scale, prospective studies should be performed in the future. Secondly, the interpretation of the optimal ETT depth was not decided upon by a single neonatologist. However, all the neonatologists used the same definition of optimal ETT depth. Thirdly, the ETT was fixed to the lip, which is movable rather than immobile. Although we secured the ETTs, there was still some deviation that is hard to avoid.

## 5. Conclusions

In conclusion, this study implies that when there is a discrepancy between the recommended ETT depth according to weight-based and age-based suggestions, the weight-based estimate should provide the optimal depth and minimize the frequency of ETT adjustments. Moreover, these results can be applied not only in AGA neonates but also in non-AGA neonates.

## Figures and Tables

**Table 1:** Initial recommended endotracheal tube insertion depth for intubation.

Gestational Age (Weeks)	Endotracheal Tube Insertion Depth (cm)	Body Weight (grams)
23–24	5.5	500–600
25–26	6.0	700–800
27–29	6.5	900–1000
30–32	7.0	1100–1400
33–34	7.5	1500–1800
35–37	8.0	1900–2400
38–40	8.5	2500–3100
41–43	9.0	3200–4200

Adapted from Kempley, S.T.; Moreira, J.W.; Petrone, F.L. Endotracheal tube length for neonatal intubation. *Resuscitation* **2008**, *77*, 369–373 [7].

**Table 2:** Baseline characteristics of the intubated neonates.

Characteristics (N = 180)	
Gestational age, weeks	33.66 ± 5.46
Term, *n* (%)	63 (35.0%)
Preterm (*n* = 117) (GA ^1^)	
34–36 weeks	29 (24.8%)
<34 weeks	88 (75.2%)
Birth weight (grams)	1986.50 ± 914.45
≧2500	65 (36.1%)
Low Birth Weight (1500–2499)	49 (27.2%)
Very Low Birth Weight (1000–1499)	29 (16.1%)
Extremely Low Birth Weight (<1000)	37 (20.6%)
Appropriate for Gestational Age ^1^	135 (75%)
Small for Gestational Age ^2^	36 (20%)
Large for Gestational Age ^3^	9 (5%)
Birth weight (Z-score ^4^)	−0.41 ± 0.98
Male, *n* (%)	111 (61.7%)
Vaginal delivery	72 (40.0%)
Apgar score at 1 min	5.4 ± 2.4
Apgar score at 5 min	7.2 ± 2.1
Reasons for intubation	
Respiratory distress syndrome, *n* (%)	83 (46.1%)
Hypoxic-ischemic encephalopathy, *n* (%)	19 (10.6%)
For operation, *n* (%)	26 (14.4%)
Sepsis, *n* (%)	13 (7.2%)
Congenital cyanotic heart disease	9 (5%)
Persistent pulmonary hypertension of newborn, *n* (%)	4 (2.2%)
Meconium aspiration syndrome, *n* (%)	3 (1.7%)
Others ^5^	23 (12.8%)
Endotracheal tube depth at 1st attempt	
Tochen’s formula (Before 2016), *n* = 96	
Correct	62 (64.6%)
Inappropriate	34 (35.4%)
After 2017, *n* = 84	
Correct	57 (67.9%)
Inappropriate	27 (32.1%)

GA: gestational age. ^1^ Appropriate for gestational age: body weight between 10–90 percentile; ^2^ Small for gestational age: body weight less than 10 percentile; ^3^ Large for gestational age: body weight more than 90 percentile; ^4^ Z-score: the standard deviation of weight-for-age. ^5^ Others: congenital diaphragm hernia, neonatal seizure, neuromuscular disorder, air leak syndrome, hypovolemic shock, and airway compression by tumor.

**Table 3:** Comparison of recommended endotracheal tube depth between two groups.

	Group 1 (N = 86)	Group 2 (N = 94)	*p* *
Sex			0.99
Male	53	58	
Female	33	36	
Age of intubation (GA ^1^ or PMA ^2^, weeks)	35.8 (29.0–39.0)	34.0 (29.1–38.0)	0.52
Body weight (gram)	2036.5 (1014.5–2846.5)	1955.0 (1280.5–2846.3)	0.81
Body height ^3^ (cm)	45.0 (36.0–49.0)	44.5 (35.8–49.0)	0.98
Head circumference ^4^ (cm)	34.5 (26.0–33.9)	31.0 (25.7–33.5)	0.71
Mode of delivery			0.27
Vaginal delivery	38	34	
Cesarean section	48	60	
Apgar score ^5^			
1 min	6.0 (4.0–8.0)	6.0 (4.0–7.0)	0.84
5 min	8.0 (6.0–9.0)	8.0 (70–9.0)	0.47
Initial ETT ^6^ depth (cm)	8.0 (7.0–9.0)	8.0 (7.0–8.5)	0.82
The ETT depth at 1st attempt			0.32
Correct	60	59	
Need revision	26	35	
Depth of adjustment (cm)	0.0 (0.0–0.0)	0.0 (0.0–0.0)	0.79
Appropriate for Gestational Age ^7^			0.11
Yes	66	62	
No	20	32	
ETT depth adjustment for Age based formula (cm)	0.5 (0.0–0.5)	0.5 (0.0–1.0)	0.008
ETT depth adjustment for BW ^8^ based formula (cm)	0.5 (0.0–0.5)	0.0 (0.0–0.5)	0.08

* The *p*-value was analyzed by the Mann–Whitney U test.^1^ GA: gestational age; ^2^ PMA: postmenstrual age: gestational age plus chronological age; ^3^ There are 5 patients with missing data in each group. ^4^ There are 2 patients with missing data in the consistent group.^5^ One neonate was delivered at home so there is no Apgar score recorded. ^6^ ETT: endotracheal tube. ^7^ Appropriate for gestational age: body weight between 10–90 percentile. ^8^ BW: body weight.

**Table 4:** Multivariable linear regression for factors associated with final endotracheal tube depth in neonates.

	Crude		Adjusted	
Variables	B ^4^ (95% CI ^5^)	*p*	B ^4^ (95% CI ^5^)	*p*
Age (GA ^1^ or PMA ^2^)	0.17 (0.16 to 0.19)	<0.001	0.04 (−0.00 to 0.08)	0.08
Body weight	0.00 (0.00 to 0.00)	<0.001	0.00 (0.001 to 0.001)	<0.001
Body length	0.12 (0.11 to 0.14)	<0.001	0.00 (−0.02 to 0.03)	0.83
Sex	0.42 (0.11 to 0.74)	<0.05	0.10 (−0.04 to 0.25)	0.16
Non-AGA ^3^	0.07 (−0.28 to 0.41)	0.71	−0.01 (−0.19 to 0.16)	0.88

^1^ GA: gestational age; ^2^ PMA: Postmenstrual age: gestational age plus chronological age; ^3^ AGA: appropriate for gestational age; ^4^ B: regression coefficients; ^5^ CI: Confidence Interval.

## Data Availability

The data presented in this study are available on request from the corresponding author. The data are not publicly available because they report private information about participants.
